# Nanostructured carriers as innovative tools for cancer diagnosis and therapy

**DOI:** 10.1063/1.5079943

**Published:** 2019-03-26

**Authors:** Chiara Martinelli, Carlotta Pucci, Gianni Ciofani

**Affiliations:** 1 Istituto Italiano di Tecnologia, Smart Bio-Interfaces, Pontedera (Pisa) 56025, Italy; 2 Department of Mechanical and Aerospace Engineering, Politecnico di Torino, Torino 10129, Italy

## Abstract

Cancer accounts for millions of deaths every year and, due to the increase and aging of the world population, the number of new diagnosed cases is continuously rising. Although many progresses in early diagnosis and innovative therapeutic protocols have been already set in clinical practice, still a lot of critical aspects need to be addressed in order to efficiently treat cancer and to reduce several drawbacks caused by conventional therapies. Nanomedicine has emerged as a very promising approach to support both early diagnosis and effective therapy of tumors, and a plethora of different inorganic and organic multifunctional nanomaterials have been *ad hoc* designed to meet the constant demand for new solutions in cancer treatment. Given their unique features and extreme versatility, nanocarriers represent an innovative and easily adaptable tool both for imaging and targeted therapy purposes, in order to improve the specific delivery of drugs administered to cancer patients. The current review reports an in-depth analysis of the most recent research studies aiming at developing both inorganic and organic materials for nanomedical applications in cancer diagnosis and therapy. A detailed overview of different approaches currently undergoing clinical trials or already approved in clinical practice is provided.

## I. INTRODUCTION

Cancer is one of the main causes of death worldwide and, according to the World Health Organization, the number of cancer-related deaths is going to increase up to approximately 13.2 million people a year by 2030.^1^ Currently, cancer treatments rely on chemotherapy, radiotherapy, and surgery. Unfortunately, these approaches are not specific, since they can attack both tumor and healthy tissues, causing adverse side effects to already debilitated patients (e.g., nausea, hair loss, weakness, and immuno-depression).

Aggressive tumors proliferate by creating new vessels in the surrounding tissues through a process termed angiogenesis. This vasculature shows several abnormalities in the number of endothelial cells and tridimensional structure, and the gaps between neighboring cells are larger than in physiological conditions, resulting in enhanced permeability.^2,3^ Moreover, tumor tissues lack an efficient lymphatic drainage system. All these phenomena are at the origin of the “enhanced permeability and retention” (EPR) effect, thanks to which some drugs can accumulate more easily around tumor tissues with respect to healthy ones.^4^ However, due to the augmented pressure at the core of the tumor mass, common drugs penetrate with many difficulties and are mostly retained at the periphery.^2,5,6^

For these reasons, there is an increasing and urgent need for designing new tools capable of improving diagnosis and reducing the severe reactions correlated with conventional therapies. Recently, researchers are putting a lot of effort in creating drugs that univocally target cancer cells and are highly bioavailable, in order to decrease the administration doses and to prevent undesired cytotoxicity and drug resistance.^7^ In the last two decades, a branch of nanotechnology, namely nanomedicine, emerged as an innovative way to exploit nanomaterials for human health, including cancer treatment.^8^ Nanomedicine allows performing early diagnosis, curing with minimal side effects, and evaluating the efficacy of the treatments in a non-invasive way.

Nanoparticles are colloidal systems very small in size (from 1 up to 1000 nm), with a high surface-to-volume ratio, and morphology and properties dependent on the components and on the preparation protocols. They can be used as therapeutic agents (magnetic nanoparticles generating hyperthermia, for instance), as drug carriers, or as contrast agents for imaging purposes. To be exploited in biomedical applications, nanomaterials must be biocompatible, well characterized, and stable *in vivo*. Nanoparticles can be easily engineered to enhance their selectivity and efficacy towards tumor cells,^9–13^ and present several advantages compared to traditional plain chemotherapeutic agents: they can (i) encapsulate hydrophobic molecules, increasing their solubility/biocompatibility and their retention time in tumoral leaky vasculature;^14–16^ (ii) be conjugated with targeting ligands for diagnostic and therapeutic purposes, improving intracellular penetration and enhancing specificity towards a selected target;^17–21^ and (iii) release the drug in a stable and controlled manner.^7,8,14,22^ Nowadays, several kinds of nanomaterials are under investigation for clinical purposes, ranging from inorganic to organic nanocarriers, and many of them have already been accepted or are under evaluation.

In this review, we will provide an overview of the main nanoparticles that are currently tested for potential exploitation in nanomedicine; applications to biological models and their current status in the clinical context will be also described, highlighting their impact as cancer nanotheranostic agents.

## II. NANOPARTICLES FOR NANOMEDICAL APPLICATIONS

**FIG. 1.**
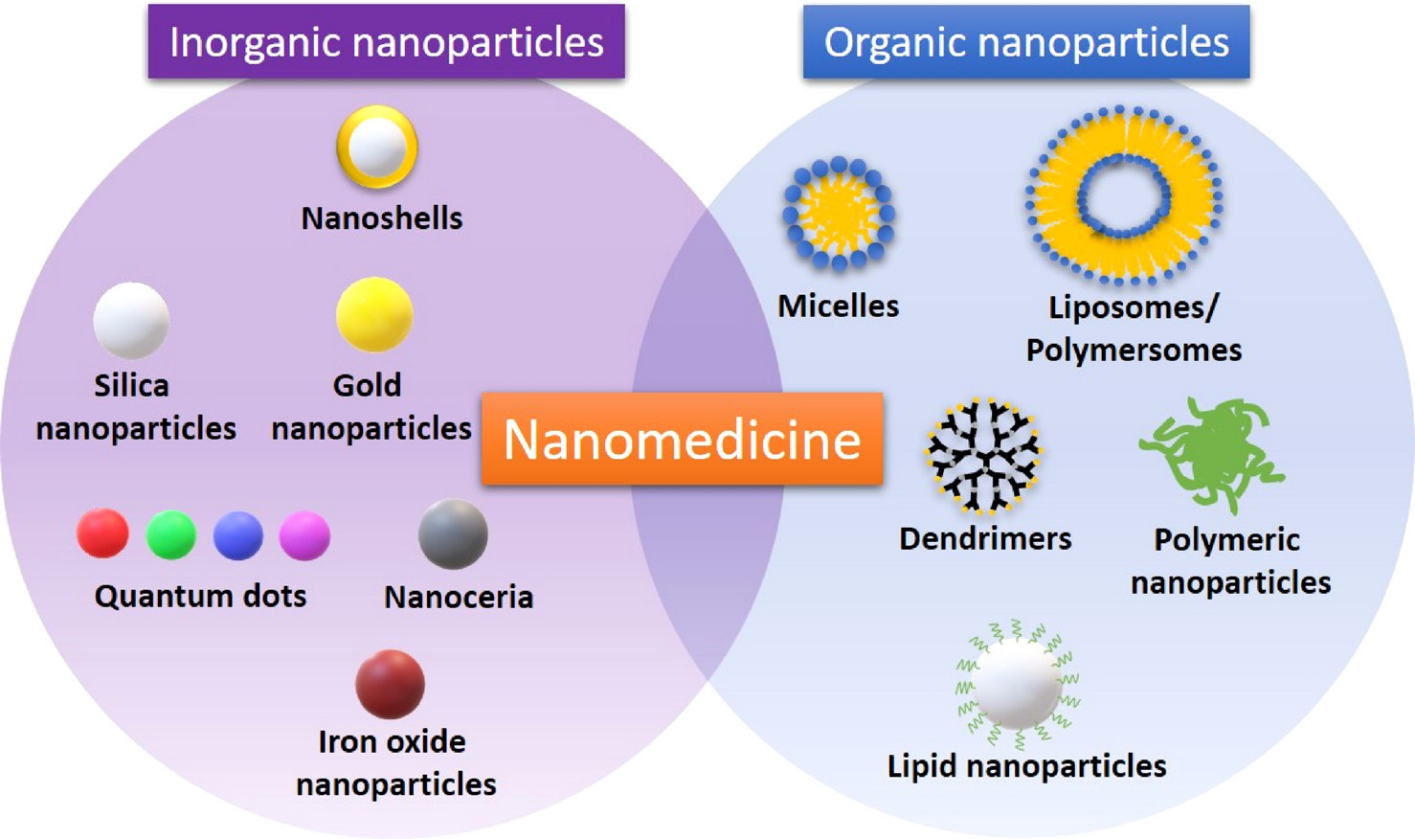
Main types of inorganic and organic nanoparticles. Nanomedicine comprises many kinds of nanovectors that can be used individually or in tandem to give the best medical performance (i.e., theranostic).

Usually, nano-biomaterials are synthesized from inorganic metals or polymers and lipids; therefore, they can be generally classified as inorganic or organic (Fig. 1).

### A. Inorganic nanoparticles

This class of nanoparticles is composed of inorganic compounds, like metal oxides or pure metals; some relevant examples are described in the following.

Nanoshells are spherical particles composed of a dielectric core (silica, in general) surrounded by a thin layer of metal. This structure makes them strongly resonate with light at specific wavelengths, and the resonance can be tuned in a large spectrum of wavelength by manipulating the shell thickness and the nanoparticle size.^23^ Nanoshells can either absorb or scatter light; absorbing nanoshells are mainly used to generate hyperthermia, whereas scattering ones are used as contrast agents.^23,24^

Silica nanoparticles have an easily tunable mesoporous structure and a high surface/volume ratio that ensure a high loading capacity and a homogeneous distribution of drugs or imaging agents. The solid framework composed of Si-O bonds is extremely resistant to degradation or external stresses. Moreover, they have lower toxicity and higher biocompatibility compared to other metal oxides.^25^

Gold nanoparticles have peculiar optical and electrical properties, low toxicity, and potential biodegradability.^19,26,27^ Moreover, gold chemistry is relatively easy, and the synthesis can be performed by following simple procedures, with good yields in terms of quantity and monodispersity. Gold nanoparticles have been investigated as a delivery system for therapeutic agents,^28^ in photodynamic therapy for the treatment of cancer,^29^ or in diagnostics to detect biomarkers for different kinds of diseases.^30^

Quantum dots (QDs) are a class of small nanoparticles (2–50 nm in diameter) consisting of a semiconducting material, with specific electronic and optical properties, due to their high surface-to-volume ratio. The final size plays an important role in these properties:^21^ for this reason, QDs can be synthesized with a core-shell structure, in order to block the size of the internal core to the desired radius. Moreover, if the shell is made of a different semiconductor with a higher band gap, QDs properties can be tuned.^31^ QDs have intense fluorescence, resistance to photobleaching, and high sensitivity for detection; thus, they are often exploited for imaging purposes. However, they can also be used as drug carriers in theranostic applications.^32^

Iron oxide nanoparticles can mainly have superparamagnetic or ferromagnetic properties, even though superparamagnetic ones are preferred in nanomedicine.^33^ In these systems, the stimulation with a magnetic field will produce the alignment of magnetic domains with the applied field. When removed, the magnetisation of superparamagnetic particles reverts to zero, and heat is released because of relaxation processes (Brownian and/or Neel relaxations);^34^ conversely, in ferromagnetic systems, the magnetisation does not spontaneously revert to zero, but a specific magnetic field (coercivity field) is needed. Heat generation is used for tumor thermal ablation through hyperthermia. Nanotherm^®^, a formulation of iron oxide nanoparticles of around 15 nm coated with aminosilane, has obtained approval for the treatment of glioblastoma.^35^

Nanoparticles of cerium oxide (nanoceria) are also under investigation for the treatment of tumors, by combining their antioxidant properties to an efficient entrapment of chemotherapeutic agents in their porous structure.^36–38^

### B. Organic nanoparticles

Liposomes are made of phospholipids, lipids, and cholesterol. Due to their amphiphilic nature, phospholipids spontaneously self-assemble in water, forming spherical structures in which the hydrophilic “head” faces towards the solvent, and the hydrophobic “tails” form the lipid bilayer. Liposomes can have one or more lipid bilayers, but they all enclose an aqueous core, mimicking the morphology of cell membranes, and they can encapsulate both hydrophilic and hydrophobic drugs.^39^ An outer layer of poly(ethylene glycol) (PEG) is often necessary to enhance their stealth stability. PEG is known to provide steric stabilization, extended blood circulation, and reduced uptake from the mononuclear phagocyte system.^40^ Doxil^®^, Myocet^®^ or DaunoXome^®^ are liposomal formulations already approved by the Food and Drug Administration (FDA) for chemotherapy.^41–43^

Polymersomes are morphological analogous of conventional liposomes, but they are composed of synthetic amphiphilic block copolymers. An amphiphilic block copolymer consists of two or more blocks of different polymers linked together by covalent bonds; one of the blocks is a hydrophilic polymer, usually PEG, and the other one can be any biocompatible polymer, such as poly(lactic acid),^44^ poly(lactic-co-glycolic acid),^45,46^ polystyrene, or polycaprolactone.^47^ Block copolymers can be designed to have specific properties in order to obtain nanoparticles with the desired features.^48^ Polymersomes possess higher stability, higher mechanical resistance, and reduced permeability compared to liposomes.^49,50^

Micelles are spherical aggregates made of amphiphilic macromolecules where the hydrophilic part is facing the solvent, while the hydrophobic tails are confined in the core. Contrary to liposomes, micelles encapsulate hydrophobic drugs in the hydrophobic core, whereas hydrophilic drugs can be adsorbed or chemically attached to the outer shell, usually made of PEG or poly(vinyl alcohol). The hydrophilic shell increases the solubility and the stability of the nanoparticles in aqueous environments.^51^ The critical micelle concentration of amphiphilic polymers is usually very low, therefore dilution *in vivo* is not an issue for the stability of the aggregates.^52^

Polymer nanoparticles are either solid spheres or nanocapsules composed by biocompatible and biodegradable polymers such as poly(lactide), poly(lactide-co-glycolide), and poly (ε-caprolactone), or natural polymers like chitosan, alginate, gelatin, and albumin.^53^ Chitosan nanoparticles are known to form electrostatic complexes with DNA, being thus very promising for non-viral gene therapy.^54,55^ Nanogels are polymeric nanoparticles where the polymers cross-link in a porous network that ensures high drug entrapment efficiency.^56^ The cross-linking can be obtained through a chemical reaction with the formation of covalent bonds, or through non-covalent interactions (physical cross-linking). In the latter case, stability *in vivo* must be carefully evaluated before final application.

Dendrimers are a class of polymers with a peculiar structure characterized by a central core—an atom or group of atoms—and multiple branches that end with several terminal functional groups.^57,58^ The branches extend symmetrically and radially from the core forming an overall globular shape. The advantage of dendrimers is that their architecture can be controlled with high precision, giving rise to well-defined and monodisperse objects. Moreover, their synthesis is extremely versatile, and either natural or synthetic polymers can be used as starting materials. Hydrophilic or hydrophobic drugs can be incorporated in the core of the dendrimers, depending on the nature of the monomers composing the macromolecule.

Solid lipid nanoparticles (SLNs) are made of lipids that are solids at body temperature (fatty acids, steroids, waxes, monoglycerides, diglycerides, or triglycerides). A small percentage of surfactants or polymeric stabilizers in the aqueous solution are needed during the preparation because of the high hydrophobicity of lipids. The kind of lipids and surfactants used in the formulation will affect the physicochemical properties of the particles.^59^ PEGylated lipids are often included in the formulation to impart steric stability and to allow for functionalization. Hydrophobic drugs are encapsulated during the fabrication, whereas hydrophilic drugs need to be either chemically attached to the components or dissolved in the hydrophilic PEG shell.^60^ Compared to liposomes, lipid nanoparticles ensure a higher drug stability and prolonged release because of their crystalline structure. Moreover, with respect to other organic nanoparticles, they do not need organic solvents during their fabrication, making them safer to use. However, the high crystallinity of solid lipid nanoparticles can cause low drug loading efficiency and/or very slow drug release profiles. For this reason, nanostructured lipid carriers (NLCs), that include one or more lipids liquid at room temperature (like oleic acid, for example), are often preferred.^61^ Depending on the amount of liquid lipid, its insertion will give rise to amorphous or partially crystalline solid matrices, increased drug release rates, and facilitated drug encapsulation during the preparation step.^62^

## III. FUNCTIONALIZATION, TARGETING AND TRIGGERED RELEASE

A current challenge in nanomedicine is the synthesis of nanoparticles that are selective for a specific target. This accomplishment would reduce the side effects of the treatment and, at the same time, would increase its efficacy. The concept of site-specific drugs was suggested by the Nobel laureate Paul Ehrlich, who developed the concept of “magic bullet” referring to drugs able to kill specific microbes without harming the rest of the body.^63^ Since then, this view has been extended to other areas of medicine.

**FIG. 2.**
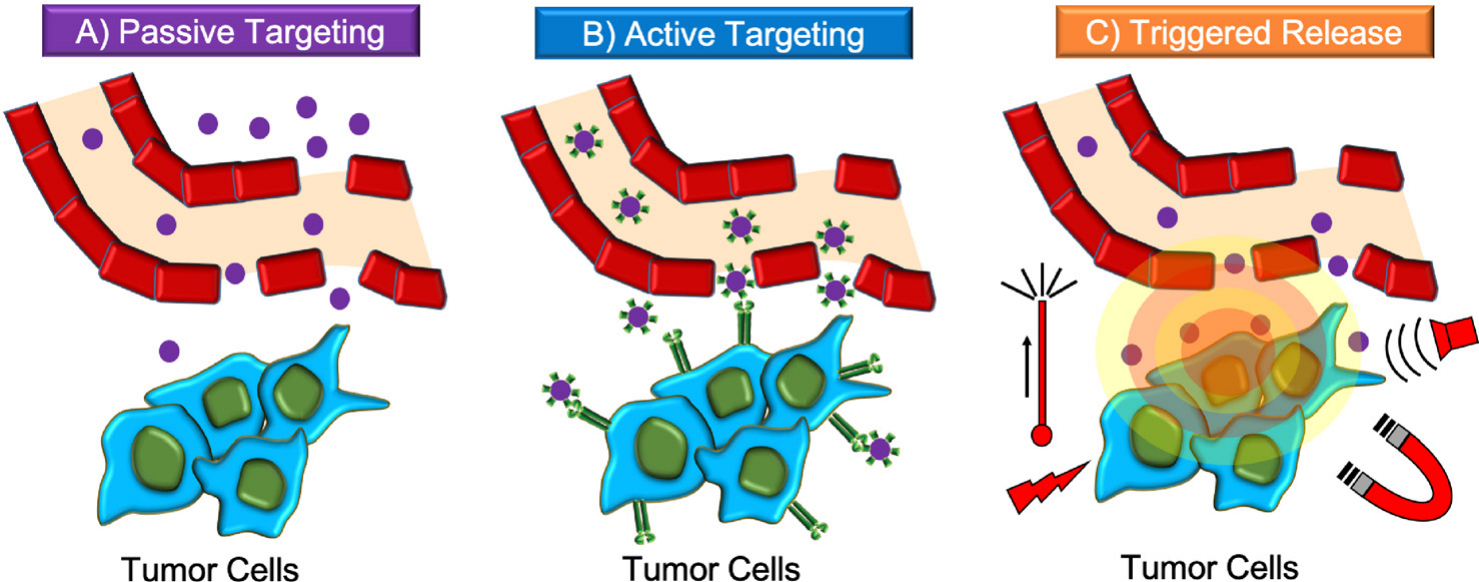
Passive targeting, active targeting, and triggered release. (a) Passive targeting relies on extravasation of nanoparticles through leaky tumor vasculature; (b) active targeting exploits surface modified nanoparticles; and (c) triggered release is based on stimuli-responsive nanoparticles.

Because of their size, nanoparticles tend to accumulate more in tumor tissues with respect to normal ones^64^ due to the enhanced permeability and retention (EPR) effect. Passive targeting relies on the fact that tumors have a leaky vasculature, different pH, and different local temperature, and are devoid of an efficient lymphatic drainage system^65^ [Fig. 2(a)]. For example, lipid-based nanovectors can reach tumor sites regardless of their surface, and can easily enter lymphatic circulation.^66^ Passive targeting, however, suffers from several limitations, such as a difficult control of the process, which may induce multi-drug resistance (MDR), a poor drug diffusion, and aspecific accumulation in liver and spleen.^67^ Targeted drug delivery has solved some of these drawbacks,^68^ allowing to specifically reach tumor cells, accumulate the vectors locally into the tumor microenvironment, and efficiently release the drug at the desired site, without perturbing healthy tissues.^69^ This can be achieved through two different approaches: (i) active targeting [Fig. 2(b)] and (ii) triggered release [Fig. 2(c)].^70^

Active targeting aims at “programming” the nanocarriers to target specific cells after extravasation. It relies on the molecular recognition (ligand-receptor or antigen-antibody interactions) of the diseased cells through specific receptors that are overexpressed on them, but that are instead normally or minimally expressed on healthy cells.^71,72^ The ligand must have a high affinity for its receptor, and the interaction must be able to trigger receptor-mediated endocytosis, after which the intracellular release can be favored by acidic pH or enzymes.^73,74^ If the affinity between the ligand and receptor is too strong, however, the penetration of the carrier might be hindered.^75^

Nanocarriers for active targeting need to be functionalized with ligands that bind specific receptors on the cell membrane.^76^ The strategies for the functionalization are different and depend on the nature of the materials involved. A ligand can be either adsorbed onto the particle surface (biotin-streptavidin interactions, for instance) or covalently bound to one of the components of the nanoparticles, normally poly(ethylene glycol). In the second case, the conjugation can occur before or after the preparation of the nanoparticles: the size of the ligand is crucial in the choice of one of these two methods. Large ligands are usually attached after the formation of the carrier, because they could alter the hydrophilic/lipophilic balance of the component, changing the condition in which the self-assembly takes place.^77^ For small ligands, instead, both ways can be exploited. The second approach, however, has some drawbacks: purification procedures, like centrifugation or filtration, may affect the stability and the features of the nanoparticles, and it is more difficult to demonstrate a successful conjugation with conventional techniques.^78–80^

Different kinds of ligands can be used to target cancer cells, and one of the simplest molecule that can be exploited at this aim is folic acid. It belongs to the vitamin B family, and is essential for nucleotide biosynthesis, while its receptor is overexpressed in several tumors, especially in ovarian and endometrial cancers.^81^ PEG-coated polymeric nanoparticles coupled to folic acid have been developed.^82^ More recently, poly(D,L-lactide-co-glycolide) (PLGA) nanoparticles carrying paclitaxel and functionalized with folic acid were able to efficiently transport the drug in Caco-2 cells, paving the way to the delivery of drugs with poor oral bioavailability.^83^ Another study demonstrated that folate-decorated chitosan nanoparticles were able to univocally deliver ligustrazine to cancer cells overexpressing folate receptors.^84^ Concerning cancer imaging, many studies have shown that superparamagnetic iron oxide nanoparticles conjugated to folic acid can be exploited as contrast agents for magnetic resonance imaging (MRI).^85–87^ The advantages of using small ligands are their low costs and easy handling. The same applies for carbohydrates, like galactose, lactose, or mannose, among others, that specifically bind to asialoglycoprotein receptors^88,89^ and to C-type lectin receptors overexpressed in cancer cell membranes.^90^ Solid lipid nanoparticles loaded with doxorubicin have been mannosylated and tested for drug release ability and cytotoxicity in A549 cells.^91^

Another category of targeting ligands includes peptides and proteins. Peptides are short chains of amino acids that can be easily synthesized to have a particular sequence. They are stable for long time and reduce undesired effects on the immune system. Moreover, being small, they do not alter the physicochemical properties of the nanoparticles.^92,93^ Cell penetrating peptides (CPPs) are often exploited to increase the permeability of the nanoparticles. However, since they are not specific for a particular receptor, they are often coupled with other ligands. Angiopep-2 is a peptide derived from the Kunitz domain of aprotinin that efficiently binds to low-density lipoprotein receptor-related protein-1 (LRP1) of endothelial cells in the blood-brain barrier (BBB). For this reason, it is often used to target cancer cells in the brain.^94^ Apolipoproteins (Apos) interact with low-density lipoprotein receptors as well, and they are used for the same purpose.^95^ Transferrin receptors (TfRs) are also overexpressed on solid tumors, especially in glioblastoma multiforme cells,^96^ and on the epithelial cells of the BBB,^97^ due to increased iron uptake required for cancer cell proliferation.

Antibodies are a special class of proteins with a typical “Y” shape, where the tips have a specific amino acid sequence called antigen-binding fragment (Fab), that univocally binds an antigen. This kind of interaction is highly specific and strong, making antibodies the most effective ligands. Even though their high molecular weight can affect the physicochemical properties of the nanoparticles or compromise the protection of the PEG layer,^98^ just a very small amount of antibody is needed to target a specific site.^79^ The production of conventional antibodies is difficult and expensive; therefore, antibody fragments containing the Fab region are often preferred, because they are safer against non-specific binding and can be easily engineered.^98,99^ Human serum albumin nanoparticles carrying loperamide have been successfully conjugated to monoclonal antibodies that specifically bind the transferrin receptor. The delivery was revealed to be efficient, and the drug was transported across the blood-brain barrier.^100^ Recent studies showed that immunoliposomes can accumulate in the brain endothelium thanks to transferrin receptor targeting.^101^

Finally, a new class of ligands is represented by small synthetic single-stranded RNA or DNA oligonucleotides (normally composed of 20–60 nucleotides), called aptamers, that can form specific shapes (helices or single-stranded loops). They are extremely versatile, and can bind different kind of targets—proteins, inorganic molecules, and cells—with a high selectivity. For this reason, they are considered an equivalent of antibodies, but their preparation is much simpler and cheaper,^102,103^ additionally to not showing any sign of toxicity.^104^

Triggered release is intended as the localized release of drugs induced by a stimulus that alters the structure of the nanocarrier.^105^ The main advantage of this type of system is that it is highly specific and can be activated “on demand” without perturbing healthy tissues. Triggers can be internal, such as variations in pH, redox conditions, and ionic strength,^106–108^ or external, such as temperature, ultrasounds, magnetic fields, and ultraviolet/near-infrared (UV/NIR) radiation. Nanocarriers can be designed to be responsive to these stimuli and to achieve enhanced release of their cargo in a precise location.^109^ Moreover, external stimuli such as local hyperthermia and UV/NIR light can enhance the permeability of blood vessels and favor deep tissue penetration.^110–112^ Finally, ultrasounds can induce release of contrast agents at the tumor site, while magnetic fields can locally drive nanocarriers, thus triggering drug release through hyperthermia.^113^

## IV. NANOCARRIERS FOR DIAGNOSIS

One of the main difficulties related to cancer diagnosis is the low sensitivity of conventional equipment. Tumors start to be detectable when they are already around 1 cm^3^ in size, a stage at which they are already able to spread in surrounding tissues, potentially creating metastatic lesions. Tissue biopsies for cancer diagnosis provide information regarding the tumor grade and its histological features, but fail to detect early stage lesions.

Recently, molecular imaging procedures have been improved to detect early stage cancer and to monitor the tumor at the genomic level in a noninvasive way, in order to predict its evolution, and to find the best personalized therapeutic strategy.^3,114^ Many innovative approaches exploit the unique features of nanoparticles like their small size, the ability to travel along human vessels, and their specificity mediated by conjugation to targeting molecules. Nanocarriers can be designed and modified to reach both cell surface proteins and intracellular molecules by means of endocytosis. The vast majority of nanomaterials used for cancer diagnosis are based on inorganic metals, like gold, silica, quantum dots, and iron oxide nanoparticles.^115^

Nanoshells have been widely exploited as imaging agents, due to their optical resonance properties and their scattering and absorption features. In particular, they have been developed as contrast agents for optical coherence tomography (OCT).^116^ Nanoshells have been also modified by antibody conjugation, in order to target specific tumor cell receptors for cancer imaging.^117^ Gold nanorods and nanocages have been successfully used as contrast agents for photoacoustic imaging *in vivo*,^118,119^ while mesoporous silica nanoparticles are currently used for optical and magnetic resonance imaging: a multifunctional nanovector has been for example developed able to encapsulate therapeutic or imaging agents and to achieve targeted delivery in cancer cells.^120^

Quantum dots represent the ideal tool for cancer imaging because of their unique absorption and emission spectra,^121^ negligible photobleaching, and stable fluorescence.^122^ They have been conjugated to streptavidin-IgG to detect extra- and intracellular molecules, and proved to be more photostable than conventional fluorophores.^123^ In a recent study, PEGylated quantum dots were conjugated to the anti-HER2 antibody and localized in specific tumor cells.^124^ QD toxicity still raises some concerns and several studies are ongoing in order to address this point. In 2012, a pilot study performed on primates demonstrated no evident toxicity up to 90 days post-injection, but further investigations are necessary to clarify the persistence of heavy metals in the body.^125^

**FIG. 3.**
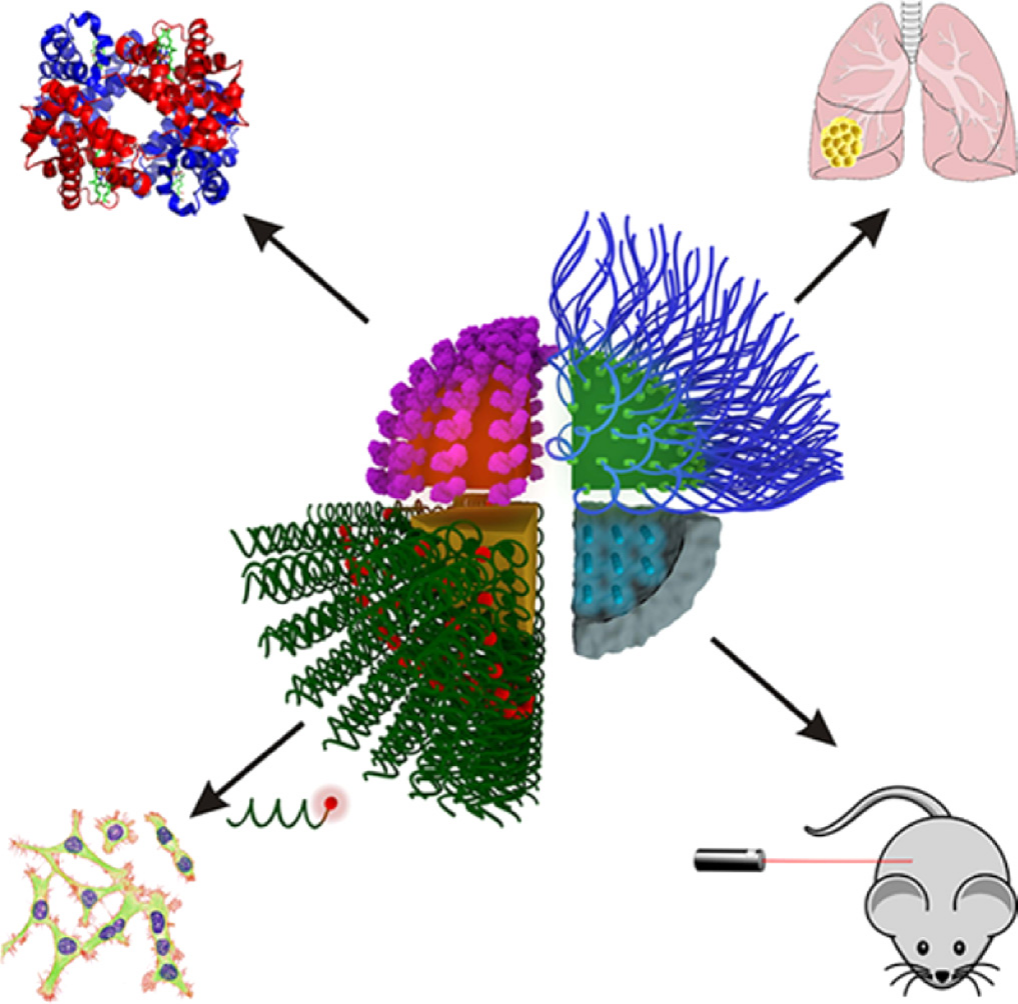
Combination of magnetic nanoparticles and fluorescent probes for targeted imaging of cancer cells and tissues. Reprinted with permission from Chinen *et al.* Chem. Rev. **115**, 72 (2015). Copyright 2015 American Chemical Society.

Superparamagnetic iron oxide nanoparticles (SPIONs) have been applied for cancer diagnosis due to their intrinsic magnetism and the possibility to be visualized by magnetic resonance imaging.^126^ Dextran-coated SPIONs have been used *in vitro* and *in vivo* as MRI contrast agents.^127,128^ They can be targeted by applying an external magnetic field and/or by functionalization with specific ligands.^129,130^ Efficient coupling to antibodies has provided localization in specific tumor models *in vivo.*^131,132^ SPIONs encapsulated within a polyacrylamide matrix and functionalized with poly(ethylene glycol) have been efficiently uptaken by tumor cells.^133^ Magnetic nanoparticles conjugated to fluorophores, chemotherapeutics or photosensitizing agents can also be exploited as theranostic devices to visualize cancer cells and simultaneously kill them^134,135^ (Fig. 3).

## V. NANOCARRIERS FOR THERAPY

A plethora of organic nanomaterials are currently under evaluation or have been already accepted for cancer therapy. Liposomes are highly biocompatible and stable, protecting cargoes from degradation. Many chemotherapeutic agents have been loaded into liposomes and delivered to cancer cells;^136,137^ moreover, liposomes have also been proposed as carriers for gene delivery and silencing, providing encouraging results both *in vitro* and *in vivo.*^138–140^ Many efforts have been devoted to enhance the drug release efficiency, by making them responsive to specific stimuli, like ultrasounds,^141,142^ light,^143^ and hyperthermia^144^ for triggered release.

Polymersomes are widely exploited for drug encapsulation and targeted delivery because of their high stability and biocompatibility. Usually, drug release is triggered upon external condition variations, like pH and redox potential, or by the presence of a magnetic field.^145^ Polymersomes made of poly(N-vinylpyrrolidone) (PVP), as a hydrophilic component, and poly(dimethylsiloxane) (PDMS), as a hydrophobic part, are a common example of these kind of systems. Recently, PVP-b-PDMS polymersomes conjugated to tumor necrosis factor alpha (TNF-alpha) have been successfully tested for *in vivo* delivery.^146^ Poly(butadiene-ethylene oxide) polymersomes loaded with paclitaxel have been demonstrated to be able to release this drug in a prolonged and stable way, with no significant cellular toxicity.^147^ Block copolymers can be also labeled with fluorescent molecules for *in vivo* studies,^148^ and loaded with photosensitizers for photodynamic therapy.^149^ Moreover, lipophilic anticancer drugs, amphiphilic dyes, and membrane proteins have been enveloped in polymersome membranes.^150–152^

Micelles are widely used as carriers of lipophilic molecules due to their high versatility and biocompatibility. Micelles can be efficiently designed in order to be responsive to different external stimuli, such as temperature^153^ and pH.^154,155^ High concentrations of drugs can be intracellularly delivered through endocytosis. Micelles have been effectively functionalized with an antibody directed against the epidermal growth factor receptor (EGFR), a glycoprotein overexpressed in several tumors.^156^ Micelles targeted to α_ν_β_3_ integrin, a regulator of cancer angiogenesis, have been reported.^157^ In recent studies, the local release of drugs from micellar nanoparticles has been achieved by exploiting the sensitivity of mitochondria to high temperatures.^158,159^ A recent preclinical study demonstrated that Genexol-PM, a paclitaxel-loaded micelle approved by the FDA, was more efficient as a radiosensitizer than plain taxol in murine models of non-small-cell lung carcinoma.^160^

Polymeric nanoparticles are highly stable in the gastrointestinal environment and allow controlled drug release.^161^ They can be functionalized for targeted delivery, and many kinds of molecules, drugs, and nucleic acids can be loaded. Chitosan nanoparticles demonstrated to be effective in releasing siRNAs both *in vitro* and *in vivo.*^162–164^ Efficient oxaliplatin delivery was obtained using hyaluronic acid-chitosan nanoparticles.^165^ Polymer nanocarriers can be also made responsive to temperature and sensitive to pH changes.^166,167^

Dendrimers possess a branched structure that can be easily modified for high specific targeting. PEGylated poly(methylmetacrylate) (PMMA) dendrimers have been exploited in B16F10 melanomas.^168^ Poly-L-lysine (PLL) dendrimers/doxorubicin complexes were able to induce anti-angiogenic responses in *in vivo* tumor models.^169^ Interestingly, dendrimers have been extensively used to deliver contrast agents for MRI imaging in glioma cells.^170^

**FIG. 4.**
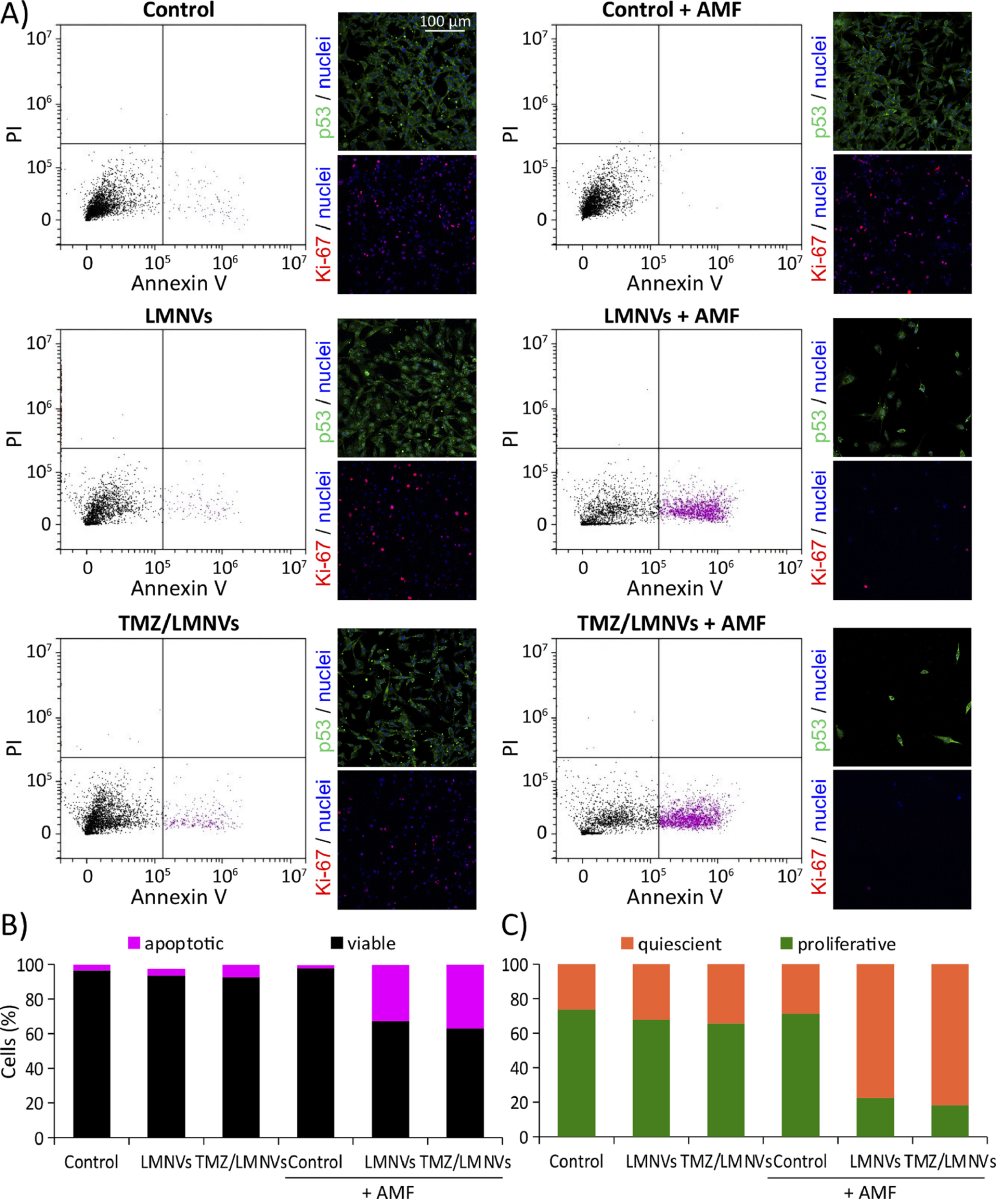
Apoptotic effect of combined treatment with hyperthermia and temozolomide (TMZ) on U-87 MG glioblastoma cells. Flow cytometer analysis shows that solid lipid nanoparticles loaded with SPIONs and TMZ (LMNVs) induce apoptosis, inhibition of proliferation, and reduction of cell number after application of an external magnetic field [(a) and (b)]. Confocal images of p53 and Ki-67 expression confirm the results obtained by flow cytometry [(a) and (c)]. Reproduced with permission from Tapeinos *et al.*, Nanoscale **11**, 72 (2019). Copyright 2019 The Royal Society of Chemistry.

Lipid nanoparticles are ideal for encapsulating hydrophobic drugs, while hydrophilic molecules can be linked to their surface. Many research studies have focused on designing solid lipid nanoparticles for delivery to cancer cells, but also as non-viral gene carrier systems.^171^ To this end, lipid-coated lipoplexes were fabricated to carry antisense oligonucleotides to the liver endothelial cells.^172^ Since lipid nanoparticles can the cross the blood-brain barrier, they are good candidates for brain tumor targeting.^173^ Etoposide encapsulated in transferrin-conjugated nanostructured lipid carriers efficiently targeted acute myelogenous leukemia cells,^174^ and induced cytotoxicity in human gastric cancer cell lines and on tumor animal models.^175^ NLCs delivering lapachone and doxorubicin were able to overcome multidrug resistance in breast cancer experimental models.^176^ Co-delivery of paclitaxel and indocyanine green was also successful in combining chemo- and photodynamic therapy *in vitro* and *in vivo.*^177^ Delivery of an epidermal growth factor receptor (EGFR) inhibitor showed evident cytotoxicity in human hepatocellular carcinoma cells.^178^ NLCs have been also modified to obtain reduced immunogenicity and longer bioavailability, and to enhance their pharmacokinetic profiles *in vivo.*^179,180^ Finally, NLCs have been recently shown to be extremely efficient tools for combining conventional chemotherapy to hyperthermia treatments, by loading their core with chemotherapeutics and SPIONs (Fig. 4).^181,182^

## VI. CANCER NANOMEDICINES: FROM BASIC RESEARCH TO CLINICAL TRANSLATION

Nanomedical devices are continuously developed and optimized in order to enhance the solubility and the bioavailability of small molecules and drugs, and to specifically deliver active drugs to diseased tissues. Approximately 250 nano-based drugs have been commercialized or are under investigation in clinical trials, and this number is constantly growing. Since the 1970s, 1 to 7 nanomedicines are FDA-approved each year.^183^ The submissions for approval include: liposomes (33%), nanocrystals (23%), emulsions (14%), iron-polymer complexes (9%), micelles (6%), and others (drug-protein complexes, drug-polymer complexes, and polymeric nanoparticles, 15%).^183^ Nanomedicine covered about 15% of the total pharmaceutical market in 2014 and is predicted to increase up to 22% in 2019 with an overall estimated business of 293.1 billion dollars by 2022, according to BCC Research.^184^ Oncology represents the largest area of nanomedicine market (about 35%), while other sectors involve neurological diseases, infections, and inflammatory and cardiovascular diseases.^185^ Several nanomedicines have been already approved by the FDA and by the European Medicines Agency (EMA) for cancer diagnosis and therapy (Table I), and many of them are currently undergoing clinical trial evaluation, possibly entering the market in the next future.

**TABLE I.** FDA and EMA-approved nano-based formulations for cancer diagnosis and therapy.

Drug	Material	Application	Indication(s)	Year(s)	References
Feridex/Endorem	Dextran-coated SPION	Diagnosis	Imaging agent	1996 (2008)	196
GastroMARK™; Umirem^®^	Silicon-coated SPION	Diagnosis	Imaging agent	2001 (2009)	197
Oncaspar^®^/Pegaspargase	PEGylated L-asparaginase	Therapy	Acute lymphoblastic	1994	198
			Leukemia		
Doxil/Caelyx™	Liposomal doxorubicin	Therapy	Kaposi's sarcoma	1995	199
			Ovarian cancer	2005	
			Multiple myeloma	2008	
DaunoXome	Liposomal daunorubicin	Therapy	Kaposi's sarcoma	1996	200
Ontak	Denileukin diftitox	Therapy	Cutaneous T-Cell lymphoma	1999	201
Myocet	Liposomal doxorubicin citrate	Therapy	Metastatic breast cancer	2000	202
Eligard^®^	PLGA [poly(lactic-co-glycolic acid)]	Therapy	Prostate cancer	2002	203
Neulasta^®^/Pegfilgrastim	PEGylated granulocyte-colony stimulating factor	Therapy	Neutropenia induced by chemotherapy	2002	204
Abraxane/ABI-007	Albumin-paclitaxel nanoparticles	Therapy	Breast cancer	2005	205
			Non-small-cell lung carcinoma	2012	
			Pancreatic cancer	2013	
Mepact^®^	Liposomal mifamurtide	Therapy	Osteosarcoma	2009	206
Nanotherm	Iron oxide	Therapy	Glioblastoma	2010	191
Marqibo^®^	Liposomal vincristine	Therapy	Acute lymphoblastic leukemia	2012	207
Onivyde^®^/MM-398	Liposomal irinotecan	Therapy	Pancreatic cancer	2015	208

Clinical translation of chemicals is subjected to many regulatory and manufactory rules before definitive approval. The approval process for a single new drug costs around 1 billion dollars and it might take 10–15 years.^186^ In spite of their simple composition, many of the chemical/physical properties of nanoparticles remain critical.^187^ *In vivo* performance has to be carefully evaluated before introducing a new product in the industrial manufacturing process.

Far in 1995, doxorubicin-loaded PEGylated liposomes (Doxil) were approved for acquired immune deficiency syndrome (AIDS)-associated Kaposi's sarcoma treatment.^188^ This formulation improved doxorubicin tolerability in patients and reduced collateral effects.^189^ After that, other liposomes developed to treat infections (Ambisome^®^), metastatic breast cancer (Myocet), and pancreatic ductal adenocarcinoma in combination with 5-fluorouracil and leucovorin (MM-398) were approved. New multifunctional liposomal nanoparticles are currently in the clinical trial phase, such as ThermoDox^®^, a formulation made of thermosensitive lipids that allows for a site-specific release of doxorubicin in response to high temperature.^190^ Other non-lipid-based FDA-approved nanoformulations include albumin-bound paclitaxel particles (Abraxane^®^), indicated for metastatic breast cancer and recently for pancreatic ductal adenocarcinoma, and an engineered protein combining interleukin-2 and diphtheria toxins for the treatment of non-Hodgkin's peripheral T-cell lymphomas (Ontak^®^). Nevertheless, other promising polymeric nanosystems such as BIND-014, a polymer micelle incorporating docetaxel, poliglumex (a paclitaxel-polyglutamic acide conjugate), and CRLX101 (a cyclodextrin-PEG copolymer encapsulating camptothecin), are under clinical trials.

Regarding inorganic nanoparticles, up until now, just some formulations containing superparamagnetic iron oxide nanoparticles (SPIONs) have been approved for clinical use for iron deficiency in anemic patients (Feraheme^®^), for the treatment of glioblastoma using local tissue hyperthermia (Nanotherm™),^191^ or as imaging agents (Feridex^®^/Endorem^®^). Other inorganic nanoparticles such as NBTXR3 (a radiosensitizer combined to radiotherapy after intravenous injection or intratumoral administration, AGulX),^192,193^ Cornell dots (silica nanoparticles for imaging applications), and a few gold nanoparticles are under clinical trials.^186^ Regarding active targeting, only a minor number of nanovectors is investigated in clinical trials, for example immunoliposomes directed against EGFR and polymeric nanoparticles for prostate cancer treatment,^194,195^ or products investigated for cardiovascular diseases or immunological tissue targeting.^17^ Actually, Ontak is the only active targeting nanomedicine that has been approved by the FDA.

## VII. CONCLUSIONS AND FUTURE PERSPECTIVES

Nowadays, nanomedicine represents one of the most promising and advanced field of biomedical research, combining nanotechnology and medicine to design agents with improved efficacy and safety for human health. In the last decade, many kinds of nanomaterials have been introduced in the biomedical field, in particular for cancer diagnosis and therapy. Ranging from inorganic to organic nanoparticles, the materials and the formulation procedures that are available for their fabrication are several, achieving high versatility, controllable size and shape, possibility to be functionalized for targeted therapy and to be loaded with several drugs and active molecules. Nevertheless, there is still room for improvement, as some aspects like cytotoxicity, immunogenicity, and low biocompatibility need to be addressed in a more extensive manner, especially for inorganic systems and for some of synthetic polymers.

In this review, we presented the most significant nanomaterials currently investigated and evaluated for clinical applications, and explored the most recent innovations in cancer diagnosis and therapy. We also discussed how nanocarriers are able to reach human organs by passive targeting, exploiting the EPR effect. However, due to a non-specific accumulation in the tumor tissues, active targeting has become the new trend in nanomedicine. By exploiting molecules overexpressed just on the cancer cell surface, in fact, active targeting has reached a high level of precision and selectivity, guaranteeing the exclusive uptake of nanoparticles in tumor cells. Future research studies will help in elucidating the molecular and the cellular mechanisms that mark healthy from pathological cells, giving a boost to the design of highly performant nano-delivery systems as tools for treating cancer. Finally, future improvements in nanomaterial characterization procedures will meet the most relevant issues required for the eventual approval of nano-drugs in clinical practice.
